# Validation of the International Classification of Functioning, Disability and Health Core Set for chronic widespread pain from the perspective of fibromyalgia patients

**DOI:** 10.1186/ar2696

**Published:** 2009-05-14

**Authors:** Robin Hieblinger, Michaela Coenen, Gerold Stucki, Andreas Winkelmann, Alarcos Cieza

**Affiliations:** 1 Department of Physical Medicine and Rehabilitation, Ludwig-Maximilian University Munich, Ziemssenstraße 1, Munich 80336, Germany; 2 ICF Research Branch of the WHO Collaborating Center for the Family of International Classifications at the German Institute of Medical Documentation and Information (DIMDI), Institute for Health and Rehabilitation Sciences, Marchioninistraße 17, Munich 81377, Germany; 3 Swiss Paraplegic Research (SPF), Guido A. Zaech Straße 4, Nottwil 6207, Switzerland

## Abstract

**Introduction:**

Functioning is recognized as an important study outcome in chronic widespread pain (CWP). The *Comprehensive ICF Core Set for CWP *is an application of the International Classification of Functioning, Disability and Health (ICF) with the purpose of representing the typical spectrum of functioning of patients with CWP. The objective of the study was to add evidence to the validation of the *Comprehensive ICF Core Set for CWP *from the patient perspective. The specific aims were to explore the aspects of functioning and health important to patients with fibromyalgia, and to examine to what extent these aspects are represented by the current version of the *Comprehensive ICF Core Set for CWP*.

**Methods:**

The sampling of patients followed the maximum variation strategy. Sample size was determined by saturation. The focus groups were digitally recorded and transcribed verbatim. The meaning condensation procedure was used for qualitative data analysis. After qualitative data analysis, the identified concepts were linked to ICF categories.

**Results:**

Thirty-three patients participated in six focus groups. Fifty-four ICF categories out of 67 categories of the *Comprehensive ICF Core Set for CWP *were reported by the patients. Forty-eight additional categories that are not covered in the *Comprehensive ICF Core Set for CWP *were raised.

**Conclusions:**

Most ICF categories of the existing version of the *Comprehensive ICF Core Set for CWP *could be confirmed from the patient perspective. However, several categories not included in the Core Set emerged and should be considered for inclusion.

## Introduction

The perspective of functioning, disability and health of the World Health Organization [1] establishes the basis for a comprehensive description of the experience of patients suffering from a determined disease. This perspective recognizes different aspects of health from a biological, individual and social perspective, providing for a coherent view of illness [2]. This holistic approach guided the development of the International Classification of Functioning, Disability and Health (ICF), which was approved by the World Health Assembly in May 2001. Since the ICF has been developed in a worldwide, comprehensive process and was endorsed by the World Health Assembly as a member of the World Health Organization Family of International Classifications, it is likely to become the generally accepted framework to describe functioning, disability and health from a bio-psycho-social perspective.

**Figure 1:**
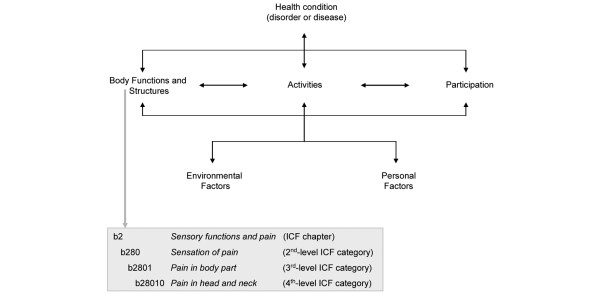
The bio-psycho-social perspective and the International Classification of Functioning, Disability and Health (ICF).

Based on the bio-psycho-social perspective, the ICF classification contains the so-called ICF components *Body Functions*, *Body Structures *and *Activities and Participation *as well as the contextual factors *Environmental *and *Personal Factors *(see Figure 1). Both functioning and disability represent the result of the interaction between *Body Functions*, *Body Structures *and *Activities and Participation *of an individual with a health condition and the contextual factors of that individual. The ICF classification contains more than 1,400 so-called ICF categories, each allotted to the named components of the classification – with the exception of the component *Personal Factors*, which has not yet been classified. Each ICF category is denoted by a code composed of a letter that refers to the components of the classification (b, *Body Functions*; s, *Body Structures*; d, *Activities and Participation*; and e, *Environmental Factors*) and is followed by a numeric code starting with the chapter number (one digit), followed by the second level (two digits) and the third and fourth levels (one digit each) (see Figure 1).

To address the issue of feasibility regarding the over 1,400 ICF categories, ICF Core Sets have been developed in a formal-decision-making and consensus-based process integrating evidence gathered from preparatory studies for a number of most burdensome, chronic health conditions. ICF Core Sets represent a selection of ICF categories out of the whole classification that can serve as minimal standards for the reporting of functioning and environmental factors for clinical studies and clinical encounters (Brief ICF Core Set) or as standards for multiprofessional, comprehensive assessment (Comprehensive ICF Core Set). Since the ICF Core Sets address aspects within all of the components of the ICF (*Body Functions*, *Body Structures*, *Activities and Participation*, *Environmental Factors*) they present a broad perspective that may reflect the whole health experience of patients.

One of the several health conditions for which ICF Core Sets were developed is chronic widespread pain (CWP). A common musculoskeletal disorder, CWP is characterized by generalized muscular pain and tenderness at multiple sites. Clinical examinations reveal no disease in joints and muscles. Fibromyalgia (FM) is one of the most severe clinical manifestations of CWP. According to the American College of Rheumatology, patients with widespread pain for at least 3 months and tenderness in 11 out of 18 tender points on digital palpation are classified as suffering FM [3]. In addition to pain, FM is characterized by fatigue, sleeping problems, mood disorder and several other symptoms as well [4]. An estimated 0.5 to 4% of the population suffers from FM [5]. Working, managing daily tasks and functioning in general can become a tremendous burden for the sufferers. Functioning represents the core of the patients' experience and is determined and influenced by their interaction with the environment and their own personal characteristics – not only by the health condition [6-9]. This is reflected by patients themselves and also by health professionals treating patients with FM. Owing to the multidimensional nature of FM, however, experts in general have recognized the difficulty of finding standardized measures, thus limiting the progress in therapeutic approaches.

The lack of standardized or validated outcome measures for FM has caused uncertainty regarding which key domains of the condition should be measured. This has been acknowledged by initiatives such as the Outcome Measures in Rheumatoid Arthritis Clinical Trials (OMERACT), the goal being to define what should be measured and how, across the spectrum of rheumatology intervention and observational studies [10]. OMERACT FM workshops have been held with the objective of standardizing and improving the quality of outcome research in FM by identifying and prioritizing domains [11,12]. The ICF can help specify OMERACT domains by serving as a conceptual model to define functioning.

The *Comprehensive ICF Core Set for CWP *describes the typical spectrum of problems in functioning among patients with CWP. Additionally, it provides an ideal basis from which to define theoretically sound models of functioning and disability in patients with CWP. The current version of the *Comprehensive ICF Core Set for CWP *includes 65 ICF categories at the second level and two ICF categories at the third level of the classification.

The *Comprehensive ICF Core Set for CWP *is now undergoing worldwide testing and validation using a number of approaches, including an international multicentre validation study and a validation from the perspective of health professionals. Since patients were not directly included in the development of the ICF Core Sets, they are now explicitly involved in the validation of ICF Core Sets to establish the patient perspective in this process. As standards of functioning and health in research and clinical practice, the ICF Core Sets have to show that they address the perspective of those who experience the disease. Since FM is a very common CWP illness with clearly defined classification criteria, we decided to focus on FM patients to validate the ICF Core Sets for CWP.

Qualitative methodology provides the possibility of exploring the perspective of those who experience a health problem; that is, the patient perspective [13,14]. Qualitative methods, especially focus groups, are now widely used and increasingly accepted in health research and health-related sciences [15-17]. The idea behind the focus group methodology is that group processes can help people explore and clarify their views [18]. The nondirective nature of focus groups allows participants to comment, explain, disagree and share experiences and attitudes [19]. The Comprehensive ICF Core Set for rheumatoid arthritis was validated recently from the patient perspective using qualitative methodology. Seventy-one out of the 76 ICF categories in this ICF Core Set were confirmed, and an additional 57 categories not covered in the ICF Core Set for rheumatoid arthritis were found [20]. Further studies for validation of ICF Core Sets from the patient perspective are currently in progress, including those for stroke, low-back pain and diabetes.

The objective of the present study was to add evidence to the validation of the *Comprehensive ICF Core Set for CWP *from the perspective of patients with FM. The specific aims were to explore the aspects of functioning and health important to patients with FM using focus group methodology and to examine to what extent these aspects are represented by the current version of the *Comprehensive ICF Core Set for CWP*.

## Materials and methods

### Design

We conducted a qualitative study with patients suffering from FM using focus groups. The study was approved by the Ethics Commission of the medical faculty of the Ludwig-Maximilian University, Munich.

### Participants

Persons with FM from three different sources – the FM day clinic of the Department of Physical Medicine and Rehabilitation of the Ludwig-Maximilian University Munich, the waiting list of the same clinic, and patients from a German self-help group of FM sufferers (Deutsche Rheuma-Liga e.V.) – were contacted and asked whether they would like to participate in the study. A sample was selected based on the maximum variation strategy [21] from the pool of patients who answered positively, the two criteria being disease duration and age. Only participants with FM diagnosed according to the American College of Rheumatology [3] and who gave written informed consent according to the Declaration of Helsinki 1996 were definitely selected.

### Sample size

The sample size was determined by saturation [22]. Saturation refers to the point at which an investigator obtains sufficient information from the field [18] (see Data analysis, Saturation of data).

### Methods

Participants filled out a patient questionnaire including sociodemographic and disease-related variables. An established topic guide with guidelines describing how to prepare and perform the focus group sessions as well as open-ended questions was applied [23]. During the focus group sessions, a visual presentation of the open-ended questions was used for better comprehension.

### Data collection

All focus groups were conducted in a nondirective manner by the same moderator (RH) and one group assistant (MC). The moderator and group assistant were psychologists with expertise in the ICF and in conducting group processes.

According to the topic guide patients were first presented with open-ended questions involving the ICF components. They were asked which FM-related problems of their body functions they were experiencing, which body structures were involved, which limitations of activities and restrictions in participation were significant to them, which environmental factors were significant to them, and which factors were barriers or facilitators for them. All ICF chapters included in the *Comprehensive ICF Core Set for CWP *were then presented one at a time. As each chapter was introduced, patients were encouraged to describe in their own words any problems they personally experienced related to each specific ICF chapter. To gain more information relevant to the participants, they were asked – after the presentation of all chapter titles of each of the ICF components – whether they thought anything important was missing (Table 1 also presents examples for the ICF chapters).

**Table 1:** Open-ended questions of the focus group, including a brief example from *Activities and Participation *component

Open-ended questions
If you think about your body and mind, what does not work the way it is supposed to?
If you think about your body, in which parts are your problems?
If you think about your daily life, what are your problems in this area?
- The next area is called Mobility. This area involves everything having to do with movement. If you think about your daily life, what are your problems in this area?^a^
- The next area is called self-care. If you think about your daily life, what are your problems in this area?^a^
- ....
- Can you think of anything else missing in this area regarding your daily life?
If you think about your environment and your living conditions, what do you find helpful or supportive?
If you think about your environment and your living conditions, what barriers do you experience?

^a^These detailed questions were used in all components for all chapters containing International Classification of Functioning, Disability and Health categories in the *Comprehensive ICF Core Set for CWP*. CWP, chronic widespread pain.

At the end of each focus group session, a summary of the main results was given back to the group to enable the participants to verify and amend emergent issues.

The focus group sessions were digitally recorded and transcribed verbatim. The assistant observed the process within the group session and took field notes according to a standardized coding schema. Field notes refer to descriptive observations of the group interaction and of the topics of discussion. To review the course of the focus group, a debriefing with the moderator and the assistant took place after each focus group.

### Data analysis

#### Qualitative analysis

The meaning condensation procedure [24] was used for the qualitative data analysis (see Table 2). In the first step, the transcripts of the focus groups were read through to gain an overview of the collected data. In the second step, the data were divided into meaning units, and the theme that dominated a meaning unit was determined. A meaning unit was defined as a specific unit of text, either a few words or a few sentences with a common theme [25]. A meaning unit division therefore did not follow linguistic grammatical rules. Rather, the text was divided where the researcher discerned a shift in meaning [24]. In the third step, the concepts contained in the meaning units were identified. A meaning unit could contain more than one concept.

**Table 2:** Scheme of the qualitative data analysis

Transcription + meaning units	Concepts	ICF categories
Moderator: The next area is called Mobility. This area involves everything having to do with movement. If you think about your daily life, what are your problems in this area?		
Patient A: Working over my head is becoming more and more difficult, like cleaning windows.	Problems working over the head	d4
	Cleaning windows	d6402
Patient B: I have to hold on to the railing and pull myself up when I go up the stairs. The next day it might be better but I really have to pull myself up to go up the stairs.	Problems going up the stairs	d4551
Patient C: After a half hour of ironing my arms hurt. Then I have to take a break. Kneeling is also a problem for me.	Pain in arm when ironing	b28014, d6403
	Problems kneeling	d4102

ICF, International Classification of Functioning, Disability and Health.

#### Linking to the ICF

The identified concepts were linked to ICF categories based on established linking rules [26,27] in a systematic and standardized way. According to these linking rules, health professionals trained in the ICF are advised to link each concept to the ICF category representing this concept most precisely.

#### Saturation of data

Saturation was defined as the point during data collection and analysis in which the linking of the concepts of two consecutive focus groups each reveal less than 5% additional ICF categories in relation to the number of ICF categories contained in the *Comprehensive ICF Core Set for CWP *that were identified in the respective previous focus group.

#### Confirmation of ICF categories

An ICF category of the *Comprehensive ICF Core Set for CWP *was regarded as confirmed if the respective ICF category had been identified after linking the information recorded from the focus groups to the ICF.

#### Additional ICF categories

All ICF categories identified in the focus groups that are included in the ICF but not in the current version of the *Comprehensive ICF Core Set for CWP *are reported as additional categories. To allow for a quick overview, only second-level ICF categories are presented in the tables.

#### Accuracy of the analysis

To ensure the accuracy of data analysis, two strategies were conducted. First, *multiple coding *– which refers to performing the qualitative analysis and the linking to the ICF of the first focus group by two health professionals. The two health professionals compared their data analysis and documented the discussion. Second, *peer review *– which refers to analysing and linking random samples of 15% of the transcribed text and 15% of the identified concepts (of the first health professional) by a second health professional. The degree of agreement between the two health professionals regarding the linked ICF categories was calculated by kappa statistic with 95%-bootstrapped confidence intervals [28]. The values of the kappa coefficient generally range from 0 to 1, where 1 indicates perfect agreement and 0 indicates no additional agreement beyond what is expected by chance alone. The Kappa analysis was performed with SAS for Windows, version 9.1 (SAS Institute Inc., Cary, NC, USA).

## Results

### Description of the focus groups

A total of 33 participants were included in six focus groups. Participants' characteristics are summarized in Table 3. The focus group sessions lasted from 70 to 115 minutes (mean 1 hour 40 minutes) including a short break.

**Table 3:** Characteristics of participants

Characteristics of participants	
Age (years)	54.4 (36 to 69)
Gender (female/male)	30/3
Disease duration (based on date of diagnosis) (years)	3.06 (0 to 17)
Living alone	9
Employment status	
Paid employment/self-employed	16
Homemaker	3
Unemployed (for health reasons)	5
Unemployed (for other reasons)	3
Pensioned	5
Pensioned due to chronic widespread pain	1

Data presented as mean (range) or *n*.

### Qualitative analysis and linking

A total of 1,686 concepts were identified in the focus groups. These concepts were linked to 247 different ICF categories of the first to the fourth levels. There were 277 concepts that could not be linked to ICF categories. Of these, 143 concepts could be allotted to the component *Personal Factors *(for example, aspects of coping, disease management) and 90 concepts were not included in the ICF classification, and therefore were defined as *not covered *(for example, time-related aspects, benefits of heat or exercise). Forty-four concepts were labelled *not definable*, which means that the concept is too unspecific to be assigned to a concrete ICF category (for example, quality of life in general).

Some concepts named by the participants were more specific than the corresponding most specific ICF category. For example, the participants reported several issues pertaining to the pain quality (pressure pain, rest pain, stabbing pain) that are not specifically covered by the existing ICF categories at that level of detail. All of these concepts referring to different qualities of pain were therefore linked to the ICF category *sensation of pain *(b280).

### Saturation of data

**Figure 2:**
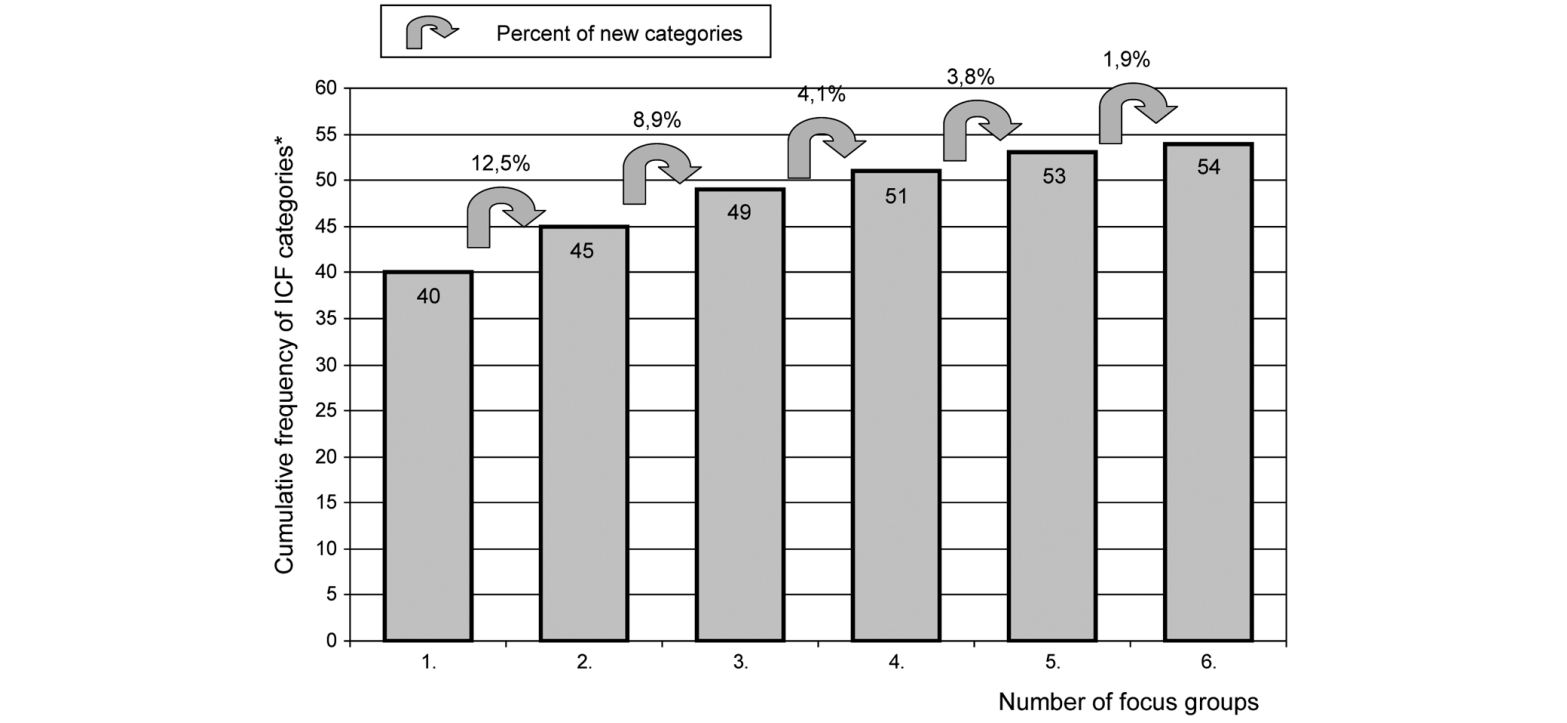
Saturation of the qualitative data in the focus groups. *Cumulative frequency of identified International Classification of Functioning, Disability and Health (ICF) categories of the *Comprehensive ICF Core Set for CWP*. CWP, chronic widespread pain.

Regarding the ICF categories of the *Comprehensive ICF Core Set for CWP*, saturation of data was reached after conducting six focus groups (see Figure 2).

### Confirmation of the *Comprehensive ICF Core Set for CWP*

In total, 54 out of the 67 ICF categories included in the *Comprehensive ICF Core Set for CWP *were confirmed by the participants: 15 out of the 23 categories of *Body Functions*, the one category of *Body Structures*, 25 out of the 27 categories of *Activities and Participation *and 13 out of the 16 categories of *Environmental Factors *(Tables 4, 5 and 6).

**Table 4:** Participants' reporting of ICF categories: *Body Functions *(b) and *Body Structures *(s)

ICF code	ICF category title	Number of linked concepts
ICF categories of the *Comprehensive ICF Core Set for CWP*		
b122	Global psychosocial functions	-
b126	Temperament and personality functions	38
b130	Energy and drive functions	51
b134	Sleep function	13
b140	Attention functions	14
b147	Psychomotor function	-
b152	Emotional functions	42
b1602	Content of thought	-
b164	Higher-level cognitive functions	-
b180	Experience of self and time functions	2
b260	Proprioceptive function	-
b265	Touch function	17
b270	Sensory functions related to temperature and other stimuli	8
b280	Sensation of pain	159
b430	Haematological system functions	-
b455	Exercise tolerance functions	18
b640	Sexual functions	-
b710	Mobility of joint functions	11
b730	Muscle power functions	19
b735	Muscle tone functions	2
b740	Muscle endurance functions	-
b760	Control of voluntary movement functions	1
b780	Sensations related to muscles and movement functions	40
s770	Additional musculoskeletal structures related to movement	1
Additional ICF categories		
b114	Orientation functions	2
b144	Memory functions	32
b210	Seeing functions	5
b220	Sensations associated with the eye and adjoining structures	4
b230	Hearing functions	12
b240	Sensations associated with hearing and vestibular function	8
b250	Taste function	4
b255	Smell function	2
b420	Blood pressure functions	6
b440	Respiration functions	2
b450	Additional respiratory functions	1
b460	Sensations associated with cardiovascular and respiratory functions	11
b510	Ingestion functions	6
b515	Digestive functions	3
b525	Defecation functions	10
b535	Sensations associated with the digestive system	3
b620	Urination functions	19
b650	Menstruation functions	2
b770	Gait pattern functions	2
b810	Protective functions of the skin	1
b820	Repair functions of the skin	1
b830	Other functions of the skin	2
b840	Sensations related to the skin	1

ICF, International Classification of Functioning, Disability and Health; CWP, chronic widespread pain.

**Table 5:** Participants' reporting of ICF categories: *Activities and Participation *(d)

ICF code	ICF category title	Number of linked concepts
ICF categories of the *Comprehensive ICF Core Set for CWP*		
d160	Focusing attention	5
d175	Solving problems	-
d220	Undertaking multiple tasks	-
d230	Carrying out daily routine	18
d240	Handling stress and other psychological demands	5
d410	Changing basic body position	44
d415	Maintaining a body position	20
d430	Lifting and carrying objects	18
d450	Walking	20
d455	Moving around	12
d470	Using transportation	1
d475	Driving	19
d510	Washing oneself	6
d540	Dressing	19
d570	Looking after one's health	2
d620	Acquisition of goods and services	4
d640	Doing housework	39
d650	Caring for household objects	5
d660	Assisting others	3
d720	Complex interpersonal interactions	2
d760	Family relationships	3
d770	Intimate relationships	9
d845	Acquiring, keeping and terminating a job	5
d850	Remunerative employment	3
d855	Non-remunerative employment	2
d910	Community life	1
d920	Recreation and leisure	30
Additional ICF categories		
d110	Watching	3
d115	Listening	5
d155	Acquiring skills	9
d163	Thinking	1
d166	Reading	3
d210	Undertaking a single task	1
d310	Communicating with – receiving – spoken messages	2
d330	Speaking	3
d360	Using communication devices and techniques	2
d440	Fine hand use	27
d445	Hand and arm use	15
d465	Moving around using equipment	5
d520	Caring for body parts	14
d630	Preparing meals	7
d740	Formal relationships	2

ICF, International Classification of Functioning, Disability and Health; CWP, chronic widespread pain.

**Table 6:** Participants' reporting of ICF categories: *Environmental Factors *(e)

ICF code	ICF category title	Number of linked concepts
ICF categories of the *Comprehensive ICF Core Set for CWP*		
e1101	Drugs	8
e310	Immediate family members	23
e325	Acquaintances, peers, colleagues, neighbours and community members	6
e355	Health professionals	9
e410	Individual attitudes of immediate family members	12
e420	Individual attitudes of friends	6
e425	Individual attitudes of acquaintances, peers, colleagues, neighbours and community members	4
e430	Individual attitudes of people in positions of authority	2
e450	Individual attitudes of health professionals	29
e455	Individual attitudes of other professionals	2
e460	Societal attitudes	-
e465	Social norms, practices and ideologies	-
e570	Social services, systems and policies	2
e575	General social services, systems and policies	-
e580	Health services, systems and policies	36
e590	Labour and employment services, systems and policies	3
Additional ICF categories		
e110	Products or substances for personal consumption	10
e115	Products and technology for personal use in daily living	39
e225	Climate	11
e320	Friends	3
e330	People in positions of authority	1
e340	Personal care providers and personal assistants	1
e415	Individual attitudes of extended family members	2
e555	Associations and organisational services, systems and policies	13
e560	Media services, systems and policies	4
e595	Political services, systems and policies	2

ICF, International Classification of Functioning, Disability and Health; CWP, chronic widespread pain.

### Additional categories

Forty-eight additional second-level ICF categories that are not included in the current version of the *Comprehensive ICF Core Set for CWP *were identified in the focus groups (Tables 4, 5 and 6). Most of the additional ICF categories stem from *Body Functions *(n = 23), followed by *Activities and Participation *(n = 15). Ten additional ICF categories reported by the participants related to *Environmental Factors*. No additional ICF categories from *Body Structures *were identified.

Twenty-two further third-level and fourth-level ICF categories emerged (data not shown), mainly from the *Activities and Participation *chapter *Mobility *(for example, d4401 *grasping*, d4153 *maintaining a sitting position*, d4552 *running*), and the *Body Functions *chapters *Mental functions *(for example, b1300 *energy level*, b1301 *motivation*, b1343 *quality of sleep*) and *Neuromusculoskeletal and movement-related functions *(for example, b7801 *sensation of muscle spasm*, b7300 *power of isolated muscles and muscle groups*).

### Accuracy of the analysis

The kappa coefficient for the agreement between the two investigators (peer review) was 0.76. The 95%-bootstrapped confidence interval was 0.70 to 0.82.

## Discussion

Most ICF categories of the current version of the *Comprehensive ICF Core Set for CWP *could be confirmed from the patient perspective by FM patients. Fifty-four ICF categories out of 67 categories of the *Comprehensive ICF Core Set for CWP *were reported by the patients. Forty-eight additional categories that are not covered in the *Comprehensive ICF Core Set for CWP *were raised. The present study also confirmed relevant outcomes of treatment in CWP and FM from the patient perspective, such as pain, fatigue, sleep disorders, psychological distress, lack of muscle power, difficulties changing and maintaining a body position, and difficulties carrying out a daily routine [29,30]. Pain proved to be the central topic reported by patients, with a total of 159 concepts linked to the ICF Core Set category *sensation of pain *(b280).

Apart from pain, the most outstanding theme reported by participants was the attitude of others regarding FM. The patients describe often feeling left alone with their illness, due to a lack of understanding and acceptance from others. Several patients reported feeling as if FM is not accepted as a legitimate illness by some doctors and healthcare professionals and is often trivialized by friends, relatives and colleagues, thus adding to the burden of pain and exhaustion. Fifty-five concepts concerning negative attitudes of others regarding the illness were linked to the corresponding ICF Core Set categories (e410, e420, e425, e430, e450, e455). Forty-six additional concepts were linked to the first-level ICF category *attitudes *(e4). Several studies report similar findings such as patients' experiences of stigma [31-34] and studies documenting controversy as to the existence, classification and acceptance of FM by healthcare professionals [35-38].

Thirteen ICF categories in the *Comprehensive ICF Core Set for CWP *were not at all mentioned by the focus groups. Most of the ICF categories belonged to *Body Functions *and included *global psychosocial functions *(b122), *psychomotor function *(b147), *content of thought *(b1602), *proprioceptive function *(b260) and *haematological system functions *(b430). Some categories were not confirmed but were linked to similar categories; for example, 18 concepts were linked to the category *carrying out daily routine *(d230) instead of *undertaking multiple tasks *(d220), and nine concepts were linked to *intimate relationships *(d770) instead of *sexual functions *(b640). Sometimes the participants made more specific statements that were linked to similar ICF categories; for example, although the ICF Core Set category *societal attitudes *(e460) was not linked, several statements were linked to categories e410 through e455 specifying individual attitudes (for example, individual attitudes of friends, colleagues, people in positions of authority, health professionals).

Forty-eight additional second-level ICF categories that are not covered in the current version of the *Comprehensive ICF Core Set for CWP *were raised. Most of the additional ICF categories belong to *Body Functions*, followed by *Activities and Participation *and *Environmental Factors*. Some of these additional ICF categories need special discussion. Several concepts deal with difficulties in cognitive functioning. Thirty-two concepts were linked to the *Body Functions *category *memory functions *(b144). The patients reported problems with short-term and long-term memory such as absorbing, storing and recalling information. Learning and applying knowledge was also perceived as challenging for the participants. Difficulties acquiring skills, thinking, hearing, listening and reading were frequently reported by the focus group participants. Poor memory performance and problems in cognitive functioning in FM sufferers have been well documented and are in accordance with other studies [39-42]. Sensations associated with hearing, such as tinnitus and dizziness, were also reported by the participants, as in other studies [43,44].

The use of the hands and arms is a further topic not included in the *Comprehensive ICF Core Set for CWP *that FM sufferers experience as very burdening. The participants reported difficulties in grasping, picking up and manipulating objects with their hands and pulling, reaching and turning or twisting the arms, making everyday activities and tasks very difficult to fulfil. Twenty-seven and 15 concepts were linked to *fine hand use *(d440) and *hand and arm use *(d445), respectively.

An additional topic found among the participants but not included in the *Comprehensive ICF Core Set for CWP *was functions of the digestive system. Such problems included difficulties with salivation, swallowing and digesting food. Urinal and intestinal irregularities were frequently reported and experienced as extremely hindering, affecting numerous activities and participation in sports and social engagements. Irritable bowel syndrome and urinary problems in FM sufferers are reported in other studies as well [45-47]. Twenty-two concepts were linked to functions related to the digestive system (b510 to b535). Some participants mentioned feeling as if fingers, hands, feet or legs were swollen although swelling was not always visible. These concepts were labelled *not definable*. Other topics concerned *Environmental Factors *not covered in the ICF classification (labelled *not covered*). Numerous patients mentioned the benefits of heat, such as using hot or warm water to sooth aching body parts. Several others recognized the importance of exercise in coping with pain and fatigue.

The characteristics of the sample in this study (gender, age, disease duration) are comparable with samples in other national [48] and international studies [49]. The validation of the ICF Core Set for rheumatoid arthritis from the patient perspective using the same approach as in the present study showed similar results. Seventy-one out of the 76 ICF categories in the ICF Core Set for Rheumatoid Arthritis were confirmed and an additional 57 categories not covered in the ICF Core Set for rheumatoid arthritis emerged [20].

It is important to mention that several strategies were used to improve and verify the trustworthiness of the data analysis. Triangulationensured the comprehensiveness of data; we included data triangulation by using two data analysts (investigator triangulation: multiple coding) [50,51]. Secondly, reflexivity was assured by conducting a research diary for the documentation of memos concerning the design, data collection and data analysis. Clear exposition was also used, establishing guidelines for conducting the focus groups (including open-ended questions), verbatim transcription, and linking rules [28]. Finally, *peer review *was included, as described earlier. The kappa coefficient of 0.76 (0.70 to 0.82) for the accuracy of the peer review is comparable with other studies reporting kappa statistics about the linking of categories [22,52,53], and can be regarded as substantial agreement.

There are some limitations of the present study that need special mention. The sample consists primarily of German residents. To establish a cross-cultural perspective we suggest that our methods be used in similar studies in other countries. Second, FM is a subtype of CWP, and may not be representative of all CWP conditions. Other ICF categories may have emerged if focus groups had been conducted with other CWP illnesses such as chronic fatigue or Gulf War syndrome. The controversy concerning the existence, classification and acceptance of FM interferes with the patients' need to be recognized and taken seriously with their illness. This may exacerbate symptoms and add to the burden of pain and exhaustion. Third, the linking process was performed by two psychologists according to established linking rules [28]. Whether other health professionals would have decided differently, however, remains unclear. Finally, we conducted six focus groups following the strategy of saturation during data analyses, with the criteria of two consecutive focus groups each revealing less than 5% additional ICF categories in relation to the number of ICF categories of the *Comprehensive ICF Core Set for CWP *identified in the respective previous focus group. Participants in a seventh focus group might still report new themes and concepts not yet addressed.

Initiatives such as the OMERACT address the challenge of standardizing and improving the quality of outcomes research by finding a common terminology and a common model of functioning and disability. The OMERACT FM workshop agreed upon the most important key domains to measure in FM. Some of the key domains mentioned are pain, patient global sense of well-being, fatigue, multidimensional aspects of functioning, sleep, depression, and treatment side effects. These domains are included in the *Comprehensive ICF Core Set for CWP*, which can in turn be used as a basis for the further specification of OMERACT domains and the development of new instruments to assess functioning for research. A further key research objective of the OMERACT initiative will be to include the patient perspective on what represents a clinically meaningful change in a domain or the syndrome as a whole. The present study can help enhance the knowledge of FM by including the patient perspective. Further research in the context of the development and confirmation of ICF Core Sets, however, is needed.

The results of the present study are comparable with the results of the validation of ICF Core Sets for rheumatoid arthritis. In line with the validation study of ICF Core Sets for CWP, most of the ICF categories included in the ICF Core Set for rheumatoid arthritis were also confirmed [20]. In addition, both studies identified numerous ICF categories from the patient perspective that were not included in the current versions of the ICF Core Set. All results of the validation studies of ICF Core Sets will be presented at an international World Health Organization conference and will be taken into account for the decision on the final versions of ICF Core Sets.

## Conclusions

It is important to consider the patient perspective for the validation of the *Comprehensive ICF Core Set for CWP*. Most ICF categories of the existing version of the *Comprehensive ICF Core Set for CWP *could be confirmed by focus groups with FM patients. Several additional categories not represented in the *Comprehensive ICF Core Set for CWP *emerged from the focus groups and should be considered for inclusion in the final version.

## Abbreviations

CWP: chronic widespread pain; FM: fibromyalgia; ICF: International Classification of Functioning, Disability and Health; OMERACT: Outcome Measures in Rheumatoid Arthritis Clinical Trials.

## Competing interests

The authors declare that they have no competing interests.

## Authors' contributions

RH conceived and organized the study and drafted the manuscript. MC participated in the performance of the focus groups and the data analysis and was involved in the peer review. GS was responsible for the overall design of the development and the validation of ICF Core Sets. AW guided the study with his input on FM. AC participated in the development of the study design and accompanied the study implementation.

## References

[B1] World Health Organization (2001). International Classification of Functioning, Disability and Health: ICF.

[B2] Stucki G, Ewert T, Cieza A (2003). Value and application of the ICF in rehabilitative medicine. Disabil Rehabil.

[B3] Wolfe F, Smythe HA, Yunus MB, Bennett RM, Bombardier C, Goldenberg DL, Tugwell P, Campbell SM, Abeles M, Clark P, Fam AG, Farber SJ, Fiechtner JJ, Franklin M, Gatter RA, Hamaty D, Lessard J, Lichtbroun AS, Masi AT, Mccain GA, Reynolds WJ, Romano TJ, Russell IJ, Sheon RP (1990). The American College of Rheumatology 1990 criteria for classification of fibromyalgia: report of the multi-center criteria committee. Arthritis Rheum.

[B4] Wolfe F, Ross K, Anderson J, Russell IJ, Hebert L (1995). The prevalence and characteristics of fibromyalgia in the general population. Arthritis Rheum.

[B5] Clauw DJ, Crofforf LJ (2003). Chronic widespread pain and fibromyalgia: what we know and what we need to know. Best Pract Res Clin Rheumatol.

[B6] Richardson JC, Ong BN, Sim J (2007). Experiencing chronic widespread pain in a family context: giving and receiving practical and emotional support. Sociol Health Illn.

[B7] Gupta A, Silman J, Ray D, Morriss R, Dickens C, MacFarlane GJ, Chiu YH, Nicholl B, McBeth J (2007). The role of psychosocial factors in predicting the onset of chronic widespread pain: results from a prospective population-based study. Rheumatology.

[B8] Sylvain H, Talbot LR (2002). Synergy towards health: a nursing intervention model for women living with fibromyalgia and their spouses. J Adv Nurs.

[B9] Henriksson CM (1994). Longterm effects of fibromyalgia on everyday life. Scand J Rheumatol.

[B10] Stucki G, Boonen A, Tugwell P, Cieza A, Boers M (2007). The World Health Organisation International Classification of Functioning, Disability and Health: a conceptual model and interface for the OMERACT process. J Rheumatol.

[B11] Mease PJ, Clauw DJ, Arnold LM, Goldenberg DL, Witter J, Williams DA, Simon LS, Strand CV, Bramson C, Martin S, Wright TM, Littman B, Wernicke JF, Gendreau RM, Crofford LJ (2005). Fibromyalgia syndrome. J Rheumatol.

[B12] Mease P, Arnold LM, Bennett R, Boonen A, Buskila D, Carville S, Chappell A, Choy E, Clauw D, Dadabhoy D, Gendreau M, Goldenberg D, Littlejohn G, Martin S, Perera P, Russell IJ, Simon L, Spaeth M, Williams D, Crofford L (2007). Fibromyalgia syndrome. J Rheumatol.

[B13] Carr AJ, Hewlett S, Hughes R, Mitchell H, Ryan S, Carr M, Kirwan J (2003). Rheumatology outcomes: the patient's perspective. J Rheumatol.

[B14] Kirwan J, Heiberg T, Hewlett S, Hughes R, Kvien T, Ahlmen M, Boers M, Minnock P, Saag K, Shea B, Suarez Almazor M, Taal E (2003). Outcomes from the Patient Perspective Workshop at OMERACT 6. J Rheumatol.

[B15] Mays N, Pope C (2000). Qualitative research in health care: assessing quality in qualitative research. BMJ.

[B16] Giacomini MK, Cook DJ (2000). Users' guides to the medical literature: XXIII. Qualitative research in health care. Are the results of the study valid?. JAMA.

[B17] Murphy E, Dingwall R, Greatbatch D, Parker S, Watson P (1998). Qualitative research methods in health technology assessment: a review of the literature. Health Technol Assess.

[B18] Kitzinger J (1995). Qualitative research: introducing focus groups. BMJ.

[B19] Powell RA, Single HM, Lloyd KR (1996). Focus groups in mental health research: enhancing the validity of user and provider questionnaires. Int J Soc Psychiatry.

[B20] Coenen M, Cieza A, Stamm TA, Amann E, Kollerits B, Stucki G (2006). Validation of the International Classification of Functioning, Disability and Health (ICF) Core Set for rheumatoid arthritis from the patient perspective using focus groups. Arthritis Res Ther.

[B21] Patton MQ (1990). Qualitative Evaluation and Research Methods.

[B22] Kvale S (1996). Interviews – An Introduction to Qualitative Research Interviewingw.

[B23] Karlsson G (1995). Psychological Qualitative Research from a Phenomenological Perspective.

[B24] Cohen J (1960). A coefficient of agreement for nominal scales. Educ Psychol Meas.

[B25] Cieza A, Brockow T, Ewert T, Amman E, Kollerits B, Chatterji S, Üstün TB, Stucki G (2002). Linking health-status measurements to the International Classification of Functioning, Disability and Health. J Rehabil Med.

[B26] Cieza A, Brockow T, Ewert T, Amman E, Kollerits B, Chatterji S, Üstün TB, Stucki G (2002). Linking health-status measurements to the International Classification of Functioning, Disability and Health. J Rehabil Med.

[B27] Cieza A, Geyh S, Chatterji S, Kostanjsek N, Üstün B, Stucki G (2005). ICF linking rules: an update based on lessons learned. J Rehabil Med.

[B28] A SAS macro for calculating bootstrapped confidence intervals about a kappa coefficient. http://www2.sas.com/proceedings/sugi22/STATS/PAPER295.PDF.

[B29] Robinson ME, Brown JL, George SZ, Edwards PS, Atchison JW, Hirsch AT, Waxenberg LB, Wittmer V, Fillingim RB (2005). Multidimensional success criteria and expectations for treatment of chronic pain: the patient perspective. Pain Med.

[B30] Wassem R, McDonald M, Racine J (2002). Fibromyalgia: patient perspectives on symptoms, symptom management, and provider utilization. Clin Nurse Spec.

[B31] Asbring P, Närvänen AL (2002). Women's experiences of stigma in relation to chronic fatigue syndrome and fibromyalgia. Qual Health Res.

[B32] Paulson M, Norberg A, Danielson E (2002). Men living with fibromyalgia-type pain: experiences as patients in the swedish health care system. J Adv Nurs.

[B33] Henriksson CM (1995). Living with continuous muscular pain – patient perspectives: part I. encounters and consequences. Scand J Caring Sci.

[B34] Söderberg S, Lundman B, Norberg A (1999). Struggling for dignity: the meaning of women's experience of living with fibromyalgia. Qual Health Res.

[B35] White KP (2004). Fibromyalgia: the answer is blowin in the wind. J Rheumatol.

[B36] Ehrlich GE (2003). Pain is real; fibromyalgia isn't. J Rheumatol.

[B37] Stahl SM (2001). Fibromyalgia: the enigma and the stigma. J Clin Psychiatry.

[B38] Wolfe F (2003). Stop using the American College of Rheumatology Criteria in the clinic. J Rheumatol.

[B39] Park DC, Glass JM, Minear M, Crofford LJ (2001). Cognitive function in fibromyalgia patients. Arthritis Rheum.

[B40] Sephton SE, Studts JL, Hoover K, Weissbecker I, Lynch G, Ho I, McGuffin S, Salmon P (2003). Biological and psychological factors associated with memory function in fibromyalgia syndrome. Health Psychol.

[B41] Sietvold H, Stiles TC, Landro NI (1995). Information processing in primary fibromyalgia, major depression and healthy controls. J Rheumatol.

[B42] Grace GM, Nielson WR, Hopkins M, Berg MA (1999). Concentration and memory deficits in patients with fibromyalgia syndrome. J Clin Exp Neuropsychol.

[B43] Bayazit YA, Gürsoy S, Özer E, Karakurum G, Madenci E (2002). Neurotologic manifestations of the fibromyalgia syndrome. J Neurol Sci.

[B44] Rosenhall U, Johansson G, Orndahl G (1996). Otoneurologic and audiologic findings in fibromyalgia. Scand J Rehabil Med.

[B45] Wolfe F, Ross K, Anderson J, Russell IJ, Hebert L (1995). The prevalence and characteristics of fibromyalgia in the general population. Arthritis Rheum.

[B46] Koziol JA (1994). Epidemiology of interstitial cystitis. Urol Clin North Am.

[B47] Sperber AD, Aczmon Y, Neumann L, Weisberg I, Shalit Y, Abu-Shakrah M, Fich A, Buskila (1999). Fibromyalgia in the irritable bowel syndrome: studies of prevalence and clinical implications. Am J Gastroenterol.

[B48] Offenbächer M, Waltz M, Schoeps P (2000). Validation of a german version of the fibromyalgia impact questionnaire (FIQ-G). J Rheumatol.

[B49] Neumann L, Buskila D (2003). Epidemiology of fibromyalgia. Curr Pain Headache Rep.

[B50] Denzin NK (1978). The Research Act: A Theoretical Introduction to Sociological Methods.

[B51] Barbour R (2001). Checklists for improving rigour in qualitative research: a case of the tail wagging the dog?. BMJ.

[B52] Landis JR, Koch GG (1977). The measurement of observer agreement for categorial data. Biometrics.

[B53] Stamm TA, Cieza A, Coenen M, Machold KP, Nell VP, Smolen JS, Stucki G (2005). Validating the international classification of functioning, disability and health comprehensive core set for rheumatoid arthritis from the patient perspective: a qualitative study. Arthritis Rheum.

